# Synthesis, Characterization and Molecular Structures of some Bismuth(III) Complexes with Thiosemicarbazones and Dithiocarbazonic Acid Methylester Derivatives with Activity against Helicobacter Pylori

**DOI:** 10.1155/MBD.1995.271

**Published:** 1995

**Authors:** Rolf Diemer, Uwe Dittes, Bernhard Nuber, Volker Seifried, Wolfgang Opferkuch, Bernhard K. Keppler

**Affiliations:** 1 Anorganisch-Chemisches Institut der Universität Heidelberg Heidelberg D-69120 Germany; 2 Ruhr-Universität Bochum Medizinische Fakultät Institut far Medizinische Mikrobiologie und Immunologie Bochum D-44780 Germany

## Abstract

The reactions of bismuth(III) nitrate pentahydrate and bismuth(III) chloride with heterocyclic thiosemicarbazones and derivatives of dithiocarbazonic acid methylester were used to synthesize the respective bismuth(III) complexes, which could be divided into five groups D-H because of their stoichiometrical properties and their molecular structures. The molecular structure and the near coordination sphere of the bismuth(III) central atom of four representative compounds were determined by single-crystal X-ray studies. Bis[1-azepanyl-4-(2-pyridyl)-2,3-diazapenta-1,3-diene-1-thiolato-N′,N^3^,S]bismuth(III) nitrate (5) belongs to group D. The two tridentate ligands and the nitrate ion surround the bismuth atom. The best description of the coordination sphere appears to be that of a distorted trigonal dodecahedron with one position occupied by the lone pair of the bismuth atom. Bis[1-azepanyl-4-(2-thienyl)-2,3-diazapenta-1,3-diene-1-thiolato-N^3^,S]bismuth(III) nitrate (9) is assigned to complex type E. Here, two deprotonated ligand molecules are coordinated to the bismuth(III) central atom as bidentate ligands. The structure of this complex can best be described as a distorted trigonal antiprism with a five-coordinated central atom. The two triangular faces are formed by the atoms S(4), N(6), O(11) and S(3), N(4) and the lone pair of the central atom. The two chelate rings are almost perpendicular to each other. Complex molecules of group F form dimeric units with bichloro-bridged bismuth atoms. The structure of di-μ-chlorobis[1-azepanyl-4-(2-pyridyl)-2,3-diazapenta-1,3-diene-1-thiolato-N′,N^3^,S-chloro]dibismuth(III) (15) can be described as two six-coordinated bismuth atoms, which are bound together via two bridging chlorine atoms. The two bismuth atoms Bi(1) and Bi(1a) and the two bridging chlorine atoms Cl(2) and Cl(2a) form the Bi_2_Cl_2_ plane. The two tridentate ligand molecules coordinate via the same atoms as shown in complex 5. In addition, they form two parallel planes, which are perpendicular to the Bi_2_Cl_2_ plane. With regard to the center of the Bi(1)-Bi(2) axis they are central point symmetrical, i.e. one pyridine ring lies above and the other beneath the Bi_2_Cl_2_ plane. Bismuth(III) chloride and pyridine-2-carboxaldehydethiosemicarbazone 1 b or 2-acetylpyridine-thiosemicarbazone 1 c form complexes of group G. Three chlorine atoms and a bidentate ligand are coordinated to the bismuth(III) central atom. The bidentate ligand bound to the central atom through the N(3) atom and the sulfur atom of the thioketo group. The structure of 18 is completely different from the structures of the bismuth(III) complexes discussed so far and was therefore assigned to group H. The bismuth central atom is coordinated with two ligands, which are bound in different ways. One of them is deprotonated. This ligand is bound to the central atom via the sulfur atom S(3) of the thiolate group and the N(5) atom. An interaction between the sulfur atom of the thiophene ring and the bismuth atom is not possible.The other ligand molecule is not deprotonated. This ligand is bound to the bismuth(III) cation merely via the sulfur atom S(1) of the thioketo group. The best description of the coordination sphere of the bismuth atom is that of a distorted square bipyramidal polyhedron. The square face is formed by the atoms S(3), N(5), Cl(1), the lone pair and the bismuth atom within. The axial positions are occupied by the atoms S(1) and Cl(2). The bond angle between S(1), Bi(1) and Cl(2) differs by about eight degrees from the value determined for a regular square bipyramidal polyhedron of 180 degrees.

Some of the newly synthesized bismuth complexes and three ligands have been tested against several strains of *Helicobacter pylori* bacteria in an agar dilution test. Almost all of the listed bismuth complexes show excellent inhibitory properties with regard to growth of *H. pylori* already at low concentrations.


### SYNTHESIS, CHARACTERIZATION AND MOLECULAR STRUCTURES OF SOME BISMUTH(III) COMPLEXES WITH THIOSEMICARBAZONES AND DITHIOCARBAZONIC ACID METHYLESTER DERIVATIVES WITH ACTIVITY AGAINST HELICOBACTER PYLORI

Rolf Diemer[1], Uwe Dittes[1], Bernhard Nuber[1], Volker Seifried[1], Wolfgang Opferkuch[2], and Bernhard K. Keppler[1]*

[1] Anorganisch-Chemisches Institut der Universität Heidelberg, D-69120 Heidelberg, Germany
[2] Ruhr-Universität Bochum, Medizinische Fakultät, Institut für Medizinische Mikrobiologie und Immunologie, D-44780 Bochum, Germany

### ABSTRACT

The reactions of bismuth(III) nitrate pentahydrate and bismuth(III) chloride with heterocyclic thiosemicarbazones and derivatives of dithiocarbazonic acid methylester were used to synthesize the respective bismuth(III) complexes, which could be divided into five groups D-H because of their stoichiometrical properties and their molecular structures. The molecular structure and the near coordination sphere of the bismuth(III) central atom of four representative compounds were determined by single-crystal X-ray studies. Bis[1-azepanyl-4-(2-pyridyl)-2,3-diazapenta-1,3-diene-1-thiolato-N',$N^3$,S]bismuth(III) nitrate (5) belongs to group D. The two tridentate ligands and the nitrate ion surround the bismuth atom. The best description of the coordination sphere appears to be that of a distorted trigonal dodecahedron with one position occupied by the lone pair of the bismuth atom. Bis[1-azepanyl-4-(2-thienyl)-2,3-diazapenta-1,3-diene-1-thiolato-$N^3$,S]bismuth(III) nitrate (9) is assigned to complex type E. Here, two deprotonated ligand molecules are coordinated to the bismuth(III) central atom as bidentate ligands. The structure of this complex can best be described as a distorted trigonal antiprism with a five-coordinated central atom. The two triangular faces are formed by the atoms S(4), N(6), O(11) and S(3), N(4) and the lone pair of the central atom. The two chelate rings are almost perpendicular to each other. Complex molecules of group F form dimeric units with bichloro-bridged bismuth atoms. The structure of di-$\mu$-chlorobis[1-azepanyl-4-(2-pyridyl)-2,3-diazapenta-1,3-diene-1-thiolato-N',$N^3$,S-chloro]dibismuth(III) (15) can be described as two six-coordinated bismuth atoms, which are bound together via two bridging chlorine atoms. The two bismuth atoms Bi(1) and Bi(1a) and the two bridging chlorine atoms Cl(2) and Cl(2a) form the $Bi_2Cl_2$ plane. The two tridentate ligand molecules coordinate via the same atoms as shown in complex 5. In addition, they form two parallel planes, which are perpendicular to the $Bi_2Cl_2$ plane. With regard to the center of the Bi(1)-Bi(2) axis they are central point symmetrical, i.e. one pyridine ring lies above and the other beneath the $Bi_2Cl_2$ plane. Bismuth(III) chloride and pyridine-2-carboxaldehydethiosemicarbazone 1 b or 2-acetylpyridinethiosemicarbazone 1 c form complexes of group G. Three chlorine atoms and a bidentate ligand are coordinated to the bismuth(III) central atom. The bidentate ligand is bound to the central atom through the N(3) atom and the sulfur atom of the thioketo group. The structure of **18** is completely different from the structures of the bismuth(III) complexes discussed so far and was therefore assigned to group H. The bismuth central atom is coordinated with two ligands, which are bound in different ways. One of them is deprotonated. This ligand is bound to the central atom via the sulfur atom S(3) of the thiolate group and the N(5) atom. An interaction between the sulfur atom of the thiophene ring and the bismuth atom is not possible.

The other ligand molecule is not deprotonated. This ligand is bound to the bismuth(III) cation merely via the sulfur atom S(1) of the thioketo group. The best description of the coordination sphere of the bismuth atom is that of a distorted square bipyramidal polyhedron. The square face is formed by the atoms S(3), N(5), Cl(1), the lone pair and the bismuth atom within. The axial positions are occupied by the atoms S(1) and Cl(2). The bond angle between S(1), Bi(1) and Cl(2) differs by about eight degrees from the value determined for a regular square bipyramidal polyhedron of 180 degrees.
Some of the newly synthesized bismuth complexes and three ligands have been tested against several strains of *Helicobacter pylori* bacteria in an agar dilution test. Almost all of the listed bismuth complexes show excellent inhibitory properties with regard to growth of *H. pylori* already at low concentrations.

### INTRODUCTION

The stomach bacterium *Helicobacter pylori* (*H. pylori*), which was discovered as late as 1983, is a pathogenic factor in the etiology of chronic gastritis and peptic ulcer.[1-5] Today, bismuth complexes such as colloidal bismuth citrate (CBS), of which the X-ray crystal structure is known, are used more frequently in the treatment of these diseases.[6-7] In the course of our research into the development of new compounds with better antibacterial activity we found that thiosemicarbazones would be suited for our intentions. Thiosemicarbazones have been known as potential antiviral and antitumor agents since 1950. The first investigations with thiosemicarbazones were carried out by Domagk, who observed considerable activity against *streptococcus*. Various thiosemicarbazones have been shown to possess an increased activity against *influenza* viruses, against a high number of *protozoa*, *plasmodium berghei* in mice and also against some types of tumor.[8-11] Coordination of some thiosemicarbazones with transition metal ions such as copper(II), nickel(II) etc. often increases their biological activities.[12]
Thiosemicarbazones coordinate as multidentate ligands both to transition metals and non-transition metals. Such complexes could be synthesized with, for example, iron, cobalt, nickel, copper, silicon and lead as central atoms.[13-15] The first bismuth(III) complex with a thiosemicarbazone derivative as ligand was published in 1992.[16] We now wish to report syntheses and single-crystal studies of bismuth(III) compounds with several thiosemicarbazones and dithiocarbazonic acid methylesters. Some complexes have also been tested for antibacterial activity against strains of *H. pylori*. Details of the syntheses, crystal structures, and tests of antibacterial activity will be presented herein.

### MATERIALS AND METHODS

The solvents were purified according to standard procedures.[17] The deuterated solvents were purchased from Aldrich or Fluka and dried over 4 Å molecular sieves. Infrared spectra were obtained on a BRUKER IFS 66 instrument with samples prepared as KBr pellets. $^{1}H$ NMR- and $^{13}C$ NMR spectra were recorded on a BRUKER AC spectrometer operating at 200 MHz. Mass spectra were recorded on a FINNIGAN MAT. 8230. Molar conductivities were determined with a WTW LF 191 conductometer operating with a Pt-electrode LS1/T-1.5. Elemental analyses were carried out in our own laboratories. The bismuth content of the compounds was determined by means of an ICP Plasma 400 from PERKIN ELMER. The following compounds were prepared according to literature procedures, their formulae and their NMR numbering schemes are listed in Scheme 1:

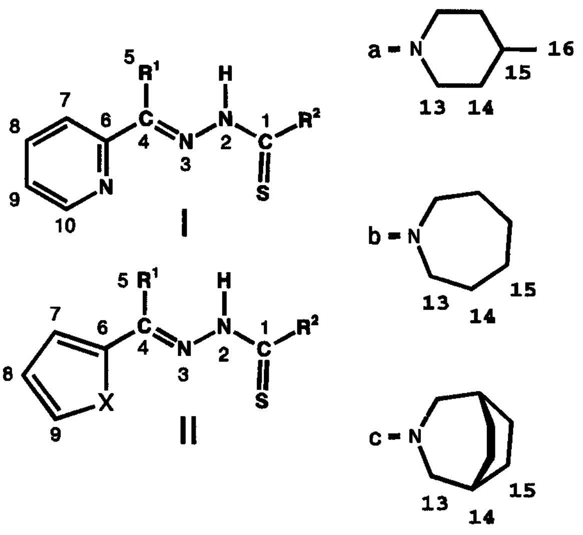

| formula I/II | $R^1$ | $R^2$ | compound |
|---|---|---|---|
| I | $-CH_3$ | $-SCH_3$ | **1 a** |
| I | H | $-NH_2$ | **1 b** |
| I | $-CH_3$ | $-NH_2$ | **1 c** |
| I | $-CH_3$ | $-N(CH_3)_2$ | **1 d** |
| I | $-CH_3$ | $-N(C_2H_5)_2$ | **1 e** |
| I | $-CH_3$ | a | **1 f** |
| I | $-CH_3$ | b | **1 g** |
| I | $-CH_3$ | c | **1 h** |
| II (X = S) | $-CH_3$ | $-SCH_3$ | **1 i** |
| II (X = S) | $-CH_3$ | b | **1 k** |
| II (X = O) | $-CH_3$ | $-SCH_3$ | **1 m** |

**Scheme 1.** Formulae of the used thiosemicarbazones and their NMR numbering schemes

3-[1-(2-Pyridyl)ethylidene]dithiocarbazonic acid methylester **1 a**,[18] pyridine-2-carboxaldehyde-thiosemicarbazone **1 b**,[14], 2-acetylpyridinethiosemicarbazone **1 c**,[19] 2-(N,N-dimethyl-aminothiocarbonyl)-1-[1-(2-pyridyl)ethylidene]hydrazine **1 d**,[10] 2-(N,N-diethylaminothiocar-bonyl)-1-[1-(2-pyridyl)ethylidene]hydrazine **1 e**,[10] 2-(4-methylpiperidinothiocarbonyl)-1-[1-(2-pyridyl)ethylidene]hydrazine **1 f**,[10] 2-(hexamethyleneiminothiocarbonyl)-1-[1-(2-pyridylethy- lidene]hydrazine **1 g**,[10] 2-[(3-azabicyclo[3.2.2]nonane-3-yl)thiocarbonyl]-1-[2-pyridyl)ethylidene]hydrazine **1 h**,[10] 3-[1-(2-thienyl)ethylidene]dithiocarbazonic acid methylester **1 i**,[18] 2-(hexamethyleneiminothiocarbonyl)-1-[1-(2-thienyl)ethylidene]hydrazine **1 k**,[10] 3-[1-(2-furyl)-ethylidene]dithiocarbazonic acid methylester **1 m**.[18] Though no $^1H$ NMR- and $^{13}C$ NMR data is listed for most of the compounds in the literature, Table 1 gives a short summary of the most important signals:

| | N2-H | H-5 | H-7 | H-8 | H-9 | H-10 | H-12 to H-15 |
|---|---|---|---|---|---|---|---|
| 1 a | 12.57 | 2.49 | 8.05 | 7.84 | 7.41 | 8.76 | $2.42^{(12)}$ |
| | | | | | | 8.56 | |
| 1 c | 10.32 | 2.36 | 8.42 | 7.76 | 7.36 | 8.55 | 8.40, 8.14 ($NH_2$) |
| 1 d | 9.62 | 2.62 | 8.04 | 8.04 | 7.5 | 8.78 | 8.78 |
| | | | | | 7.38 | 8.57 | |
| 1 f | 9.72 | 2.61 | 7.85 | 7.97 | 7.52 | 8.72 | 4.98 $4.57^{(13e)}$ $1.66^{(14e)}$ |
| | | | | | 7.38 | | 3.12 $2.95^{(13a)}$ $1.15^{(14a)}$ |
| | | | | | | | $1.66^{(15)}$ $0.92^{(16)}$ |
| 1 g | 9.47 | 2.61 | 7.84 | 7.99 | 7.5 | 8.73 | $4.03^{(13)}$ $1.76^{(14)}$ $1.48^{(15)}$ |
| | N2-H | H-5 | H-7 | H-8 | H-9 | H-12 | H-13 |
| 1 i | 12.56 | 2.63 | 7.68 | 7.27 | 7.79 | 2.53 | |
| 1 k | 13.3 | 2.69 | 7.91 | 7.33 | 8.13 | 3.96 | 1,76 |
| | 9.42 | 2.29 | 7.45 | 7.05 | 7.56 | 3.85 | |
| 1 m | 12.34 | 2.28 | 7.04 | 6.63 | 7.86 | 2.48 | |

**Table 1.** $^1H$-NMR Signals ($\delta$, DMSO-$d_6$) of the ligands 1 a, 1 c-d and 1 f-m

General procedure A: 5.0 mmol of the ligand are dissolved in methanol (50 ml), water (40 ml) and acetone (120 ml) under reflux. 2.3 mmol (1.12 g) of finely powdered $Bi(NO_3)_3$ 5 $H_2O$ are then added to the clear, light yellow solution and heated under reflux until a clear solution is obtained. When bismuth nitrate is added, the colour of the solution turns into a dark yellow. The hot solution is filtered and slowly evaporated within 30-40 minutes to give a final volume of 60 ml. Then the solution is stored at room temperature for an hour. The resulting precipitate is filtered off, washed twice with a mixture of water/methanol (1:1, 10 ml), then with ether (20 ml) , and finally dried over $P_4O_{10}$ under vacuum.

General procedure B: 4.0 mmol of the ligand are dissolved in acetone (100 ml), methanol (50 ml) and water (40 ml) under reflux. 3.5 mmol (1.1 g) of $BiCl_3$, dissolved in methanol (10 ml) and conc. HCl (0.3 ml), are added to the boiling reaction mixture within three minutes. The mixture is heated under reflux until the orange solution is completely clear. The hot solution is filtered and worked up as described in procedure A. Already during the distillation, a strongly coloured yellow precipitate forms.

General procedure C: 4.0 mmol of the ligand are dissolved in methanol (150 ml) and water (30 ml) under reflux. 3.8 mmol (1.2 g) of $BiCl_3$, dissolved in conc. HCl (2 ml) and methanol (50 ml) are added to the boiling reaction mixture within 10 minutes. The colour of the mixture turns into a deep yellow. After three minutes, a yellow precipitate forms, which is worked up as usual.

**(1) Bis[1-amino-4-(2-pyridyl)-2,3-diazabuta-1,3-diene-1-thiolato-N',N$^3$,S]bismuth(III) nitrate** was prepared with the ligand 1 b by procedure A. Yield: 1.25 g (86%), m.p. 250°C (dec.). Anal. Calc. (found) for $C_{14}H_{14}BiN_9O_3S_2$: C, 26.72 (26.81); H, 2.24 (2.32); N, 20.03 (19.88); S, 10.19 (10.26). $^1H$ NMR ($\delta$, ppm, DMSO-$d_6$, 20°C): 8.87 (d, J = 5 Hz, 2 H, H-9), 8.72 (s, 2 H, H-4), 8.10 (dd, J = 8 Hz, J =5 Hz, 2 H, H-7), 7.82 (d, J = 5 Hz, 2 H, H-6), 7.58 (s, 4 H, $NH_2$), 7.54 (m, 2 H, H-8). $^{13}C$ NMR ($\delta$, ppm, DMSO-$d_6$, 20°C): 174.7 (1-C), 149.5 (5-C),

149.1 (9-C), 147.3 (4-C), 139.3 (7-C), 126.1 (8-C), 124.9 (6-C). IR (KBr, $cm^{-1}$): 3460 m, 3354 m, 3279 s, 3162 s ($\nu_{N-H}$); 1457 vs ($\nu_{C=N-N=C}$) UV (MeOH, $\lambda_{max}$, nm): 384.7, 293.5, 248.5 $\Lambda_M$ (DMF): 74.4 [$Scm^2 mol^{-1}$].

**(2) Bis[1-dimethylamino-4-(2-pyridyl)-2,3-diazapenta-1,3-diene-1-thiolato-N',N$^3$,S]bismuth(III) nitrate** was prepared with the ligand **1 d** by procedure A. Yield: 1.15 g (70%), m.p. 210°C (dec.). Anal. Calc. (found) for $C_{20}H_{26}BiN_9O_3S_2$: C, 33.66 (33.57); H, 3.67 (3.66); N, 17.67 (17.44); S, 8.99 (9.12). $^1$H NMR ($\delta$, ppm, DMSO-$d_6$, 20°C): 8.93 (d, J = 5 Hz, 2H, H-10), 8.11 (dd, J = 8 Hz, J = 5 Hz, 2 H, H-8), 8.04 (d, J = 8 Hz, 2 H, H-7), 7.56 (dd, J = 5 Hz, J = 8 Hz, 2 H, H-9), 3.33 (s, 12 H, H-13), 2.57 (s, 6 H, H-5). IR (KBr, $cm^{-1}$): 2923 w ($\nu_{C(13)-H}$), 1391 vs ($\nu_{C=N-N=C}$). UV (MeOH, $\lambda_{max}$, nm): 397.7, 302.2, 252.9. $\Lambda_M$ (DMF): 66.5 [$Scm^2 mol^{-1}$].

**(3) Bis[1-diethylamino-4-(2-pyridyl)-2,3-diazapenta-1,3-diene-1-thiolato-N',N$^3$,S]bismuth(III) nitrate** was prepared with the ligand **1 e** by procedure A. Yield: 1.32 g (75%), m.p. 220°C (dec.). Anal. Calc. (found) for $C_{24}H_{34}BiN_9O_3S_2$: C, 37.45 (37.44); H, 4.45 (4.50); N, 16.38 (16.30); S, 8.33 (8.35). $^1$H NMR ($\delta$, ppm, DMSO-$d_6$, 20°C): 8.93 (d, J = 5 Hz, 2 H, H-10), 8.10 (dd, J = 8 Hz, J = 5 hz, 2 H, H-8), 8.02 (d, J = 8 Hz, 2 H, H-7), 7.54 (dd, J = 8 Hz, J = 5 Hz, 2 H, H-9), 3.70 (q, J = 7 Hz, 8H, H-13), 2.58 (s, 6H, H-5), 1.08 (t, J = 7 Hz, 12H, H-14). IR (KBr, $cm^{-1}$): 2977 m, 2928 m, 2868 m ($\nu_{C(13)-H}$), 1394 vs ($\nu_{C=N-N=C}$). UV (MeOH, $\lambda_{max}$, nm): 395.5, 316.9, 257.2. $\Lambda_M$ (DMF): 62.5 [$Scm^2 mol^{-1}$].

**(4) Bis[1-(4-methylpiperidino)-4-(2-pyridyl)-2,3-diazapenta-1,3-diene-1-thiolato-N',N$^3$,S]bismuth (III) nitrate** was prepared with the ligand **1 f** by procedure A. Yield: 1.55 g (82%), m.p. 205°C (dec.). Anal. Calc. (found) for $C_{28}H_{38}BiN_9O_3S_2$: C, 40.92 (40.84); H, 4.66 (4.85); N, 15.34 (15.25); S, 7.80 (7.68). $^1$H NMR ($\delta$, ppm, DMSO-$d_6$, 20°C): 8.93 (d, J = 5 Hz, 2 H, H-10), 8.12 (dd, J = 8 Hz, J = 5 Hz, 2 H, H-8), 8.04 (d, J = 8 Hz, 2 H, H-7), 7.56 (dd, J = 8 Hz, J = 5 Hz, 2 H, H-9), 4.61 (m, 4 H, 13-$H_e$), 3.03 (m, 4 H, 13-$H_a$), 2.56 (s, 6 H, H-5), 1.61 (m, 6 H, $14_e$-H, 15-H), 1.00 (m, 4 H, 14-$H_a$), 0.87 (d, J = 6 Hz, 6 H, H-16), (e = equatorial, a = axial). IR (KBr, $cm^{-1}$): 2942 m, 2920 m, 2864 m ($\nu_{C(13)-H}$), 1430 vs ($\nu_{C=N-N=C}$). UV (MeOH, $\lambda_{max}$, nm): 399.8, 306.6, 252.9. $\Lambda_M$ (DMF): 62.5 [$Scm^2 mol^{-1}$].

**(5) Bis[1-azepanyl-4-(2-pyridyl)-2,3-diazapenta-1,3-diene-1-thiolato-N',N$^3$,S]bismuth(III) nitrate** was prepared with the ligand **1 g** by procedure A. Yield: 1.35 g (71%), m.p. 233°C (dec.). Anal. Calc. (found) for $C_{28}H_{38}BiN_9O_3S_2$: C, 40.92 (40.93); H, 4.66 (4.72); Bi, 25.43 (25.70); N, 15.34 (15.23); S, 7.80 (7.75). $^1$H NMR ($\delta$, ppm, DMSO-$d_6$, 20°C): 8.93 (d, J = 5 Hz, 2H, H-10), 8.12 (dd, J = 8 Hz, J = 5 Hz, 2 H, H-8), 8.02 (d, J = 8 Hz, 2 H, H-7), 7.55 (dd, J = 5 Hz, J = 8 Hz, 2 H, H-9), 3.79 (m, 8 H, H-13), 2.57 (s, 6 H, H-5), 1.66 (m, 8 H, H-14), 1.45 ($s_b$, 8 H, H-15). $^{13}$C NMR ($\delta$, ppm, DMSO-$d_6$, 20°C): 170.4 (1-C), 154.4 (4-C), 151.7 (6-C), 148.7 (10-C), 139.4 (8-C), 124.4 (9-C), 123.2 (7-C), 50.5 (13-C), 26.0 (14,15-C), 15.1 (5-C). IR (KBr, $cm^{-1}$): 2925 s, 2825 m ($\nu_{C(13)-H}$), 1417 vs ($\nu_{C=N-N=C}$). UV (MeOH, $\lambda_{max}$, nm): 403.1, 305.5, 253.4. MS (FD): m/z = 759 [$M^+$-$NO_3$]. $\Lambda_M$ (DMF): 62.3 [$Scm^2 mol^{-1}$].

**(6) Bis[1-(3-azabicyclo[3.2.2]nonane-3-yl-4-(2-pyridyl)-2,3-diazapenta-1,3-diene-1-thiolato-N',-N$^3$,S] bismuth(III) nitrate (6)** was prepared with the ligand **1 h** by procedure A. Yield: 1.62 g (80%), m.p. 247°C (dec.). Anal. Calc. (found) for $C_{32}H_{42}BiN_9O_3S_2$: C, 43.98 (44.05); H, 4.84 (4.91); N, 14.43 (14.47); S, 7.34 (7.44). $^1$H NMR ($\delta$, ppm, DMSO-$d_6$, 20°C): 8.92 (d, J =

5 Hz, 2 H, H-10), 8.11 (dd, J = 8 Hz, J = 5 Hz, 2 H, H-8), 8.02 (d, J = 8 Hz, 2 H, H-7), 7.54 (dd, J = 5 Hz, J = 8 Hz, 2 H, H-9), 4.04 (m, 8 H, H-13), 2.56 (s, 6 H, H-5), 1.93 (m, 4 H, H-14), 1.57 (m, 16 H, H-15). IR (KBr, $cm^{-1}$): 2929 m, 2885 m, 2858 m ($\nu_{C(13)-H}$), 1430 vs ($\nu_{C=N-N=C}$). UV (MeOH, $\lambda_{max}$, nm): 402.8, 306.0, 254.3. $\Lambda_M$ (DMF): 64.0 [$Scm^2 mol^{-1}$].

**(7) Bis[1-methylthio-4-(2-pyridyl)-2,3-diazapenta-1,3-diene-1-thiolato-N',N³,S]bismuth(III) nitrate** was prepared with the ligand 1 a by procedure A.Yield: 1.37 g (83%), m.p. 200°C (dec.). Anal. Calc. (found) for $C_{18}H_{20}BiN_7O_3S_4$: C, 30.04 (29.98); H, 2.80 (2.91); Bi, 29.04 (29.00); N, 13.63 (13.55); S, 17.82 (17.58). $^1$H NMR ($\delta$, ppm, DMSO-$d_6$, 20°C): 9.02 (d, J = 5 Hz, 2 H, H-10), 8.30-8.20 (m, 4 H, H-7,8), 7.77 (m, 2 H, H-9), 2.77 (s, 6 H, H-13), 2.61 (s, 6 H, H-5). IR (KBr, $cm^{-1}$): 1444 vs ($\nu_{C=N-N=C}$). UV (MeOH, $\lambda_{max}$, nm): 371.6, 316.3, 231.2. $\Lambda_M$ (DMF): 66.6 [$Scm^2 mol^{-1}$].

**(8) Bis[1-amino-4-(2-pyridyl)-2,3-diazapenta-1,3-diene-1-thiolato-N',N³,S]bismuth(III) nitrate.** 5.15 mmol of 1 c are dissolved in methanol (70 ml) and water (30 ml) under reflux. 2.5 mmol (1.2 g) of finely powdered $Bi(NO_3)_3$ * 5 $H_2O$ are added to this solution. The reaction mixture is heated under reflux for 20 minutes. A solution of 5.0 mmol (0.2 g) NaOH in water (8 ml) is slowly added and the mixture is heated under reflux for another 5 minutes. After filtration of the hot solution, the clear orange-yellow solution is evaporated at 50°C to a final volume of 70 ml and worked up as usual. Yield: 0.85 g (52%), m.p. 220°C (dec.). Anal. Calc. (found) for $C_{16}H_{18}BiN_9O_3S_2$: C, 29.23 (29.07); H, 2.76 (2.93); Bi, 31.78 (30.50); N, 19.17 (19.14); S, 9.75 (9.99). $^1$H NMR ($\delta$, ppm, DMSO-$d_6$, 20°C): 8.95 (d, J = 5 Hz, 2 H, H-10), 8.10 (dd, J = 8 Hz, J = 5 Hz, 2 H, H-8), 8.00 (d, J = 8 Hz, 2 H, H-7), 7.56 (dd, J = 5 Hz, J = 8 Hz, 2 H, H-9), 7.42 (s, 4 H, $NH_2$), 2.54 (s, 6 H, H-5). $^{13}$C NMR ($\delta$, ppm, DMSO-$d_6$, 20°C): 170.5 (1-C), 153.9 (4-C), 151.6 (6-C), 148.6 (10-C), 139.4 (8-C), 124.6 (9-C), 123.4 (7-C), 15.2 (5-C). IR (KBr, $cm^{-1}$): 3387 m, 3290 m, 3187 s, ($\nu_{N-H}$); 1453 vs ($\nu_{C=N-N=C}$) UV (MeOH, $\lambda_{max}$, nm): 379.8, 292.4, 249.6 $\Lambda_M$ (DMF): 65.7 [$Scm^2 mol^{-1}$].

**(9) Bis[1-azepanyl-4-(2-thienyl)-2,3-diazapenta-1,3-diene-1-thiolato-N³,S]bismuth(III) nitrate.** 5.0 mmol (1.41 g) of 1 k are dissolved in methanol (200 ml) under reflux. A suspension of 1.7 mmol (0.83 g) of finely powdered $Bi(NO_3)_3 \cdot 5 H_2O$ in methanol (200 ml) is added to the boiling reaction mixture in small portions to obtain a clear orange solution. After storage at room temperature for two hours, orange-brown crystals separate, which are worked up as usual. They are dried in the drying pistol at 100°C / 1 mbar. The crystal lattice of the resulting orange-brown crystals contains one molecule methanol per complex molecule. Yield: 1.08 g (76%), m.p. 176-180°C (dec.). Anal. Calc. (found) for $C_{26}H_{36}BiN_7O_3S_4$: C, 37.54 (37.42); H, 4.36 (4.37); Bi, 25.12 (25.70); N, 11.79 (11.75); S, 15.42 (15.59). $^1$H NMR ($\delta$, ppm, DMSO-$d_6$, 20°C): 7.97 (d, J = 5 Hz, 2 H, H-9), 7.80 (d, J = 4 Hz, 2 H, H-7), 7.23 (t, J = 4 Hz, 2 H, H-8), 3.97 (m, 8 H, H-12), 2.69 (s, 6 H, H-5), 1.76 (m, 8 H, H-13), 1.49 (m, 8 H, H-14). $^{13}$C NMR ($\delta$, ppm, DMSO-$d_6$, 20°C): 168.5 (1-C), 149.9 (4-C), 135.8 (6-C), 132.5 (7-C), 132.3 (9-C), 126.6 (8-C), 52.5 (12-C), 27.4 (13-C), 26.2 (5-C), 23.5 (14-C). IR (KBr, $cm^{-1}$): 2925 m, 2825 w ($\nu_{C(13)-H}$), 1432 s ($\nu_{C=N-N=C}$). UV (MeOH, $\lambda_{max}$, nm): 365.7, 290.3, 251.2, 211.6. MS (FD): m/z = 769 [$M^+$-$NO_3$]. $\Lambda_M$ (DMF): 58.8 [$Scm^2 mol^{-1}$].

**(10) Bis[1-methylthio-4-(2-thienyl)-2,3-diazapenta-1,3-diene-1-thiolato-N³,S]bismuth(III) nitrate.** 5.0 mmol (1.15 g) of 1 i are dissolved in methanol (120 ml) under reflux. A solution of 2.3 mmol (1.12 g) of finely powdered $Bi(NO_3)_3 \cdot 5 H_2O$ in methanol (25 ml) and conc. $HNO_3$

(0.15 ml) is added to the boiling reaction mixture within one minute. On adding the bismuth nitrate solution, a voluminous yellow-orange precipitate forms immediately. The reaction mixture is heated for three minutes under reflux; then the hot suspension is filtered and the precipitate is worked up as described earlier. Yield: 1.25 g (74%), m.p. 170°C (dec.). Anal. Calc. (found) for $C_{16}H_{18}BiN_5O_3S_6$: C, 26.34 (26.42); H, 2.49 (2.47); Bi, 28.64 (28.50); N, 9.60 (9.60); S, 26.36 (26.22). $^1$H NMR ($\delta$, ppm, DMSO-$d_6$, 20°C): 8.09 (d, J = 5 Hz, 2 H, H-9), 7.91 (d, J = 4 Hz, 2 H, H-7), 7.25 (dd, J = 4 Hz, J = 5 Hz, 2 H, H-8), 2.78 (s, 6 H, H-12), 2.73 (s, 6 H, H-5). IR (KBr, cm$^{-1}$): 1384 vs ($\nu_{C=N-N=C}$). UV (MeOH, $\lambda_{max}$, nm): 344.5, 267.5, 212.7. $\Lambda_M$ (DMF): 47.6 [Scm$^2$ mol$^{-1}$].

**(11) Bis[1-methylthio-4-(2-furyl)-2,3-diazapenta-1,3-diene-1-thiolato-N$^3$,S]bismuth(III) nitrate.** 5.0 mmol (1.07 g) of **1 m** are dissolved in methanol (80 ml) under reflux. A suspension of 2.1 mmol (1.02 g) finely powdered $Bi(NO_3)_3 \cdot 5\ H_2O$ in methanol (60 ml) is added to the boiling reaction mixture, then the clear orange solution is treated as described in procedure **A**. Yield: 0.78 g (53%), m.p. 170°C (dec.). Anal. Calc. (found) for $C_{16}H_{18}BiN_5O_5S_4$: C, 27.55 (27.57); H, 2.60 (2.73); Bi, 29.95 (30.80); N, 10.04 (9.81); S, 18.39 (18.19). $^1$H NMR ($\delta$, ppm, DMSO-$d_6$, 20°C): 7.99, 6.74 (d, J = 1 Hz, 2 H, H-9), 7.69, 7.34 (d, J = 4 Hz, 2 H, H-7), 6.87, 6.63 (dd, J = 4 Hz, J = 1 Hz, 2 H, H-8), 2.63, 2.45 (s, 6H, H-12), 2.55, 2.41 (s, 6 H, H-5). IR (KBr, cm$^{-1}$): 1462 vs ($\nu_{C=N-N=C}$). MS (FD): m/z = 635 [M$^+$-NO$_3$]. $\Lambda_M$ (DMF): 60.6 [Scm$^2$ mol$^{-1}$].

**(12) Di-$\mu$-chlorobis[chloro(1-dimethylamino-4-(2-pyridyl)-2,3-diazapenta-1,3-diene-1-thiolato-N',N$^3$,S)]dibismuth(III)** was prepared with the ligand **1 d** by procedure **B**. Yield: 1.53 g (87%), m.p. 270°C (dec.). Anal. Calc. (found) for $C_{20}H_{26}Bi_2Cl_4N_8S_2$: C, 23.97 (24.13); H, 2.61 (2.71); Cl, 14.15 (14.10); N, 11.18 (11.03); S, 6.40 (6.35). $^1$H NMR ($\delta$, ppm, DMSO-$d_6$, 20°C): 9.08 (d, J = 5 Hz, 2 H, H-10), 8.17 (dd, J = 8 Hz, J = 5 Hz, 2 H, H-8), 8.08 (d, J = 8 Hz, 2 H, H-7), 7.63 (dd, J = 5 Hz, J = 8 Hz, 2 H, H-9), 3.40, 3.24 (s, 12 H, H-13), 2.57 (s, 6 H, H-5). IR (KBr, cm$^{-1}$): 2925 w ($\nu_{C(13)-H}$), 1395 vs ($\nu_{C=N-N=C}$). UV (MeOH, $\lambda_{max}$, nm): 418.3, 333.1, 291.4, 256.1. $\Lambda_M$ (DMF): 7.5 [Scm$^2$ mol$^{-1}$].

**(13) Di-$\mu$-chlorobis[chloro(1-diethylamino-4-(2-pyridyl)-2,3-diazapenta-1,3-diene-1-thiolato-N',-N$^3$,S)]dibismuth(III)** was prepared with the ligand **1 e** by procedure **B**. Yield: 1.65 g (89%), m.p. 247 - 251°C. Anal. Calc. (found) for $C_{24}H_{34}Bi_2Cl_4N_8S_2$: C, 27.23 (27.29); H, 3.24 (3.27); Cl, 13.40 (13.81); N, 10.59 (10.55); S, 6.05 (6.07). $^1$H NMR ($\delta$, ppm, DMSO-$d_6$, 20°C): 9.08, 8.94 (d, J = 5 Hz, 2 H, H-10), 8.17 (dd, J = 8 Hz, J = 5 Hz, 2 H, H-8), 8.06 (d, J = 8 Hz, 2 H, H-7), 7.62 (m, J = 5 Hz, 2 H, H-9), 3.85, 3.70 (q, J = 7 Hz, 8 H, H-13), 2.58 (s, 6 H, H-5), 1.18, 1.07 (t, J = 7 Hz, 12 H, H-14). IR (KBr, cm$^{-1}$): 2973 m, 2931 m, 2869 w ($\nu_{C(13)-H}$), 1393 vs ($\nu_{C=N-N=C}$). UV (MeOH, $\lambda_{max}$, nm): 418.8, 329.9, 290.8, 257.2. $\Lambda_M$ (DMF): 8.0 [Scm$^2$ mol$^{-1}$].

**(14) Di-$\mu$-chlorobis[chloro(1-(4-methylpiperidino)-4-(2-pyridyl)-2,3-diazapenta-1,3-diene-1-thiolato-N',N$^3$,S)]dibismuth(III)** was prepared with the ligand **1 f** by procedure **B**. Yield: 1.75 g (90%), m.p. 247 - 250°C. Anal. Calc. (found) for $C_{28}H_{38}Bi_2Cl_4N_8S_2$: C, 30.28 (30.41); H, 3.45 (3.51); Cl, 12.77 (13.04); N, 10.09 (10.00); S, 5.77 (5.67). $^1$H NMR ($\delta$, ppm, DMSO-$d_6$, 20°C): 9.09, 8.95 (d, J = 5 Hz, 2 H, H-10), 8.18 (dd, J = 8 Hz, J = 5 Hz, 2 H, H-8), 8.07 (d, J = 8 Hz, 2 H, H-7), 7.64 (dd, J = 5 Hz, J = 8 Hz, 2 H, H-9), 4.78 (m, 4 H, 13-H$_e$) 3.18 (m, 4 H, 13-H$_a$), 2.57 (s, 6 H, H-5), 1.65 (m, 6 H, 14$_e$H-,15), 1.15 (m, 4 H, 14-H$_a$), 0.93 (d,

6 H, H-16). IR (KBr, $cm^{-1}$): 2947 m, 2923 m, 2864 m ($\nu_{C(13)-H}$), 1432 vs ($\nu_{C=N-N=C}$). UV (MeOH, $\lambda_{max}$, nm): 418.8, 334.8, 291.9, 257.2. $\Lambda_M$ (DMF): 8.2 [$Scm^2 mol^{-1}$].

**(15) Di-μ-chlorobis[1-azepanyl-4-(2-pyridyl)-2,3-diazapenta-1,3-diene-1-thiolato-N',N³,S-chloro]dibismuth(III)** was prepared with the ligand **1 g** by procedure B. Yield: 1.78 g (91%), m.p. 245 - 248°C. Anal. Calc. (found) for $C_{28}H_{38}Bi_2Cl_4N_8S_2$: C, 30.28 (30.33); H, 3.45 (3.43); Bi, 37.64 (36.80); Cl, 12.77 (12.94); N, 10.09 (9.87); S, 5.77 (5.79). $^1H$ NMR (δ, ppm, DMSO-$d_6$, 20°C): 9.08 (d, J = 5 Hz, 2 H, H-10), 8.16 (m, J = 8 Hz, 2 H, H-8), 8.05 (d, J = 8 Hz, 2 H, H-7), 7.63 (m, J = 5 Hz, 2 H, H-9), 4.00 (m, 8 H, H-13), 2.58 (s, 6 H, H-5), 1.76 (m, 8 H, H-14), 1.51 (m, 8 H, H-15). IR (KBr, $cm^{-1}$): 2929 m, 2854 m ($\nu_{C(13)-H}$), 1421 vs ($\nu_{C=N-N=C}$). UV (MeOH, $\lambda_{max}$, nm): 419.4, 331.5, 293.6, 256.1. MS (FD): m/z = 1073, 1075, 1077 ($M^+$-Cl). $\Lambda_M$ (DMF): 8.6 [$Scm^2 mol^{-1}$].

**(16) Di-μ-chlorobis[1-(3-azabicyclo[3.2.2]nonane-3-yl)-4-(2-pyridyl)-2,3-diazapenta-1,3-diene-1-thiolato-N',N³,S-chloro]dibismuth(III)** was prepared with the ligand **1 h** by procedure B. Yield: 1.80 g (88%), m.p. 243 - 246°C. Anal. Calc. (found) for $C_{32}H_{42}Bi_2Cl_4N_8S_2$: C, 33.06 (32.77); H, 3.64 (3.70); Cl, 12.20 (12.27); N, 9.64 (9.42); S, 5.51 (5.40). $^1H$ NMR (δ, ppm, DMSO-$d_6$, 20°C): 9.08, 8.95 (d, J = 5 Hz, 2 H, H-10), 8.17 (dd, J = 8 Hz, J = 5 Hz, 2 H, H-8), 8.05 (d, J = 8 Hz, 2 H, H-7), 7.62, 7.54 (dd, J = 5 Hz, J = 8 Hz, 2 H, H-9), 4.17, 4.03 (m, 8 H, H-13), 2.57 (s, 6 H, H-5), 2.08, 1.99 (m, 4 H, H-14), 1.60 (m 16 H, H-15). IR (KBr, $cm^{-1}$): 2931 m, 2905 m, 2860 m ($\nu_{C(13)-H}$), 1430 vs ($\nu_{C=N-N=C}$). UV (MeOH, $\lambda_{max}$, nm): 422.6, 338.0, 291.9, 257.7. $\Lambda_M$ (DMF): 9.6 [$Scm^2 mol^{-1}$].

**(17) Di-μ-chlorobis[chloro(1-methylthio-4-(2-pyridyl)-2,3-diazapenta-1,3-diene-1-thiolato-N',-N³,S)]dibismuth(III)** was prepared with the ligand **1 a** by procedure B with a slight modification. The ligand was dissolved in acetone (80 ml), methanol (50 ml) and water (25 ml). Yield: 1.66 g (94%), m.p. 240°C (dec.). Anal. Calc. (found) for $C_{18}H_{20}Bi_2Cl_4N_6S_4$: C, 21.44 (21.68); H, 2.00 (2.05); Bi, 41.45 (41.30); Cl, 14.06 (14.05); N, 8.33 (8.32); S, 12.72 (12.95). $^1H$ NMR (δ, ppm, DMSO-$d_6$, 20°C): 9.19 (d, J = 5 Hz, 2 H, H-10), 8.25 (m, 4 H, H-7H-,8), 7.79, 7.72 (dd, J = 5 Hz, J = 8 Hz, 2 H, H-9), 2.76, 2.72 (s, 6 H, H-13), 2.67, 2.57 (s, 6 H, H-5). IR (KBr, $cm^{-1}$): 1437 vs ($\nu_{C=N-N=C}$). UV (MeOH, $\lambda_{max}$, nm): 385.2, 329.9, 291.4, 242.0. $\Lambda_M$ (DMF): 7.8 [$Scm^2 mol^{-1}$].

**(18) [1-Azepanyl-4-(2-thienyl)-2,3-diazapenta-1,3-diene-1-thiolato-N³,S]-[1-azepanyl-4-(2-thienyl)-2,3-diazapent-3-ene-1-thiolketo-S]dichlorobismuth(III).** 4.0 mmol (1.13 g) of **1 k** are dissolved in methanol (110 ml) under reflux. 2.0 mmol (0.63 g) of $BiCl_3$ in methanol (50 ml) and conc. HCl (0.3 ml) are added to the boiling solution within three minutes. Then the solution is worked up as described in procedure C. Yield: 0.92 g (55%), m.p. 174 - 177°C. Anal. Calc. (found) for $C_{26}H_{37}BiCl_2N_6S_4$: C, 37.10 (37.11); H, 4.43 (4.49); Bi, 24.83 (24.90); Cl, 8.42 (8.60); N, 9.98 (9.99); S, 15.24 (15.25). $^1H$ NMR (δ, ppm, DMSO-$d_6$, 20°C): 9.42 (s, 1H, NH), 8.12, 7.73, 7.54 (d, 2 H, H-9), 7.91, 7.49 (d, 2 H, H-7), 7.33, 7.19, 7.03 (dd, 2 H, H-8), 3.95-3.85 (m, 8 H, H-12), 2.69, 2.63, 2.28 (s, 6 H, H-5), 1.77 (m, 8 H, H-13), 1.52 (m, 8 H, H-14). IR (KBr, $cm^{-1}$): 2925 s, 2851 m ($\nu_{C(13)-H}$), 1432 vs ($\nu_{C=N-N=C}$), 1267 s ($\nu_{C=S}$) UV (MeOH, $\lambda_{max}$, nm): 366.2, 290.8, 251.8, 213.3. MS (FD): m/z = 804, 806 ($M^+$-HCl). $\Lambda_M$ (DMF): 3.0 [$Scm^2 mol^{-1}$].

**(19) Pyridine-2-carboxyaldehydethiosemicarbazone-N[3],S-trichlorobismuth(III)** was prepared with the ligand **1 b** by procedure C. Yield: 1.75 g (93%), m.p. 240°C (dec.). Anal. Calc. (found) for $C_7H_8BiCl_3N_4S$: C, 16.96 (17.13); H, 1.63 (1.77); Cl, 21.46 (22.36); N, 11.31 (11.30); S, 6.47 (6.03). $^1$H NMR ($\delta$, ppm, DMSO-$d_6$, 20°C): 11.78 (s, 1H, NH), 8.60 (d, J = 5 Hz, 1 H, H-9), 8.45, 8.29 (s, 2 H, $NH_2$), 8.15 (d, J = 8 Hz, 1 H, H-6), 8.08 (s, 1 H, H-4), 7.97 (dd, J = 8 Hz, J = 5 Hz, 1 H, H-7), 7.50 (dd, J = 5 Hz, J = 8 Hz, 1 H, H-8). $^{13}$C NMR ($\delta$, ppm, DMSO-$d_6$, 20°C): 178.5 (1-C), 151.9 (5-C), 147.9 (9-C), 140.2 (4-C), 138.6 (7-C), 124.6 (8-C), 121.4 (6-C). IR (KBr, $cm^{-1}$): 3322 m, 3258 s, 3167 s ($\nu_{N-H}$); UV (MeOH, $\lambda_{max}$, nm): 398.8, 322.3, 290.3, 249.6. $\Lambda_M$ (DMF): 74.5 [$Scm^2mol^{-1}$].

**(20) 2-Acetylpyridinethiosemicarbazone-N[3],S-trichlorobismuth(III)** was prepared with the ligand **1 c** by procedure C. Yield: 1.78 g (92%), m.p. 250°C (dec.). Anal. Calc. (found) for $C_8H_{10}BiCl_3N_4S$: C, 18.86 (19.37); H, 1.97 (1.99); Cl, 20.87 (20.85); N, 10.99 (10.91); S, 6.29 (6.18). $^1$H NMR ($\delta$, ppm, DMSO-$d_6$, 20°C): 10.35 (s, 1H, NH), 8.56 (d, J = 5 Hz, 1 H, H-10), 8.42, 8.16 (s, 2 H, $NH_2$), 8.43 (d, J = 8 Hz, 1 H, H-7), 7.80 (dd, J = 8 Hz, J = 5 Hz, 1 H, H-8), 7.39 (dd, J = 5 Hz, J = 8 Hz, 1 H, H-9), 2.37 (s, 3 H, H-5). IR (KBr, $cm^{-1}$): 3393 m, 3248 s, 3170 s ($\nu_{N-H}$). UV (MeOH, $\lambda_{max}$, nm): 404.2, 316.3, 289.2, 253.4. $\Lambda_M$ (DMF): 77.3 [$Scm^2mol^{-1}$].

The crystals needed for X-ray crystal structure analysis are produced as follows: 1 g of the bismuth complex is dissolved at 40°C in as little methanol as possible. The clear solution is heated within 5 minutes under reflux and is then filtered off at a high temperature. The solution is cooled down to room temperature and closed with a semipermeable membrane so the methanol cannot evaporate but slowly. The mixtures are generally stored at room temperature for a period of two to four weeks. The crystals are filtered off and dried. The crystals of 9 were immediately put into an impermeable matrix.

**Activity of some bismuth(III) complexes against *H. pylori*.** A number of bismuth(III) complexes with thiosemicarbazone and dithiocarbazonic acid methylester derivatives as ligands and some ligands themselves have been screened in an agar dilution test for their activity against *H. pylori*. Five different strains of *H. pylori* are incubated for 24 hours under microaerophilic conditions (5 % $CO_2$) in brain heart infusions at 37 °C and supplemented with yeast extract (0.25 %), hermin (1 %) and horse serum (10 %). The bismuth complexes, having been dissolved in DMSO, are diluted with water in a geometrical dilution series to the corresponding concentrations. These solutions are given onto blood agar plates (4 % sheep blood) in the order of falling concentrations 512 - 2 $\mu$g/ml. 20 $\mu$l of the bacterial suspension are spot inoculated on the blood agar plates containing the dilutions of the bismuth complexes. After absorption of the suspensions, the inoculum plates are incubated at 37 °C in anaerobic jars with Anaerocult C (Merck No. 16275), about 8-10 % by volume $CO_2$ and 5-7 % by volume $O_2$ for 5-6 days. The lowest concentration of the bismuth complexes leading to complete inhibition of bacterial growth was determined (in $\mu$g/ml agar). Reading of these minimal inhibitory concentration (MIC) results is rather easy, reliable and reproducible with the agar-dilution method whereas the broth-dilution leads to deviating results due to difficulties interpreting the turbidity. Growth control experiments for nonsupplemented blood agar plates and blood agar plates containing DMSO (2 %) for all strains are included for all strains. Table 2 shows the MIC values for some representative bismuth complexes and free ligands:

| Compound | NCTC 11637 | H.p. 8981 | H.p. 16 | H.p. 43 | H.p. 72 |
|---|---|---|---|---|---|
| **1 f** | 25 | 10 | 1 | 10 | 10 |
| **4** | 10 | 10 | 0.5 | 10 | 10 |
| **1 g** | 10 | 5 | < 0.2 | 2.5 | 10 |
| **5** | 10 | 10 | 5 | 10 | 10 |
| **1 h** | 25 | 10 | < 0.2 | 5 | 25 |
| **6** | 25 | 10 | 10 | 10 | 25 |
| **16** | 100 | 50 | 50 | 100 | 100 |
| **2** | 25 | 10 | 5 | 10 | 10 |
| **13** | 25 | 10 | 5 | 5 | 10 |

**Table 2.** Minimal inhibitory concentrations (MIC) in µg/ml of some bismuth complexes against *H. pylori*.

Bismuth citrate, which is the preferred bismuth complex in the treatment of gastritic infections, shows MIC values between 16 - $\geq$ 64 µg/ml. With the exception of complex **16**, all the listed bismuth complexes show excellent inhibitory properties with regard to growth of *H. pylori* already at low concentrations. By comparison of the obtained MIC values for free and coordinated ligand molecules, the resulting bismuth(III) complexes are superior to the ligand molecules themselves, which could be seen after the calculation of the relative ligand concentration (RLC). As an example of this method, we compare the MIC values for the ligand **1 h** and the synthesized complex **6**, obtained by reaction of **1 h** with bismuth(III) nitrate pentahydrate, and the chlorobridged dimeric compound **16**. Against the *H.pylori* strain NCTC 11637, the MIC value for **1 h** is 25µg/ml or 82.7 nmol/ml of **1 h**. The MIC value for **6** is 25µg/ml or 28.6 nmol/ml of **6**. Since the complex **6** contains two moles of ligand molecules per formula unit, the resulting RLC is 57.2 nmol/ml for the ligand **1 h**. Though dissociation of the complex could not be observed under the conditions of the agar dilution test, the antibacterial activity of the bismuth complex **6** depends not only upon the ligand's activity itself. In relation to compound **16**, the RLC is 172.0 nmol/ml, so this compound is less effective against H.p. bacteria than the ligand. The new bismuth compounds could be used instead of bismuth citrate in the triple-therapy, which consists of a bismuth compound, amoxicillin and metronidazole as an alternative to the combination of the ATP-ase-inhibitor opremazole with either amoxicillin or clarithromycin.[20,21] Both therapeutic regimes lead to an eradication rate of about 85-90 % and have been proven to be the most successful regimes in many different studies.

The structures of the bismuth(III) complexes **5**, **9**, **15**, and **18** could be elucidated by X-ray crystal structure analyses. The crystal was mounted on a glass fibre and placed on a Siemens-Stoe (AED II) diffractometer using a graphite-monochromated Mo-$K_\alpha$ radiation ($\lambda$ = 71.073 pm).[22] The structure was solved by conventional Patterson and difference Fourier methods using the SHELXTL-Plus program package.[23] The function minimized during full-matrix-least-squares refinement was $\Sigma w^{1/2}(|F_o-F_c|)$ with weights $w = 1/\sigma^2(|F_o|)$. Neutral atom scattering factors and anomalous dispersion corrections for the non-hydrogen atoms were taken from Ref.[24]. A selection of bond distances and angles involving the bismuth atoms is given in Tables 3-4, the fractional atomic coordinates are given in Tables 5-8:

| atoms | 5 | 9 | 15 | 18 |
|---|---|---|---|---|
| Bi(1)-S(1) | 2.584 (4) | | 2.583 (3) | 2.992 (3) |
| Bi(1)-S(2) | 2.654 (4) | | | |
| Bi(1)-S(3) | | 2.565 (4) | | 2.527 (3) |
| Bi(1)-S(4) | | 2.561 (3) | | |
| Bi(1)-N(1) | 2.711 (10) | | 2.501 (8) | |
| Bi(1)-N(2) | 2.471 (8) | | 2.355 (8) | |
| Bi(1)-N(4) | | 2.438 (10) | | |
| Bi(1)-N(5) | 2.649 (9) | | | 2.518 (8) |
| Bi(1)-N(6) | 2.581 (8) | 2.445 (11) | | |
| Bi(1)-O(11) | | 2.889 (11) | | |
| Bi(1)-O(13) | 2.731 (11) | | | |
| Bi(1)-Cl(1) | | | 2.585 (3) | 2.630 (4) |
| Bi(1)-Cl(2) | | | 2.791 (3) | 2.605 (4) |

**Table 3.** Selected Bond Distances (Å)

| atoms | 5 | 15 | atoms | 9 | 18 |
|---|---|---|---|---|---|
| S(1)-Bi(1)-S(2) | 92.3(1) | | S(3)-Bi(1)-S(4) | 96.0(1) | |
| S(2)-Bi(1)-N(1) | 86.1(3) | | S(3)-Bi(1)-N(4) | 73.2(2) | |
| S(1)-Bi(1)-N(2) | 71.9(3) | 74.8(2) | S(4)-Bi(1)-N(4) | 83.2(2) | |
| S(2)-Bi(1)-N(2) | 73.9(3) | | S(3)-Bi(1)-N(6) | 81.2(2) | |
| N(1)-Bi(1)-N(2) | 63.0(3) | 67.0(3) | S(4)-Bi(1)-N(6) | 73.7(2) | |
| N(1)-Bi(1)-S(1) | | 141.7(2) | N(4)-Bi(1)-N(6) | 143.3(3) | |
| S(1)-Bi(1)-N(5) | 73.4(2) | | Cl(1)-Bi(1)-Cl(2) | | 97.0(1) |
| S(2)-Bi(1)-N(5) | 129.5(3) | | Cl(1)-Bi(1)-S(1) | | 84.3 (1) |
| N(1)-Bi(1)-N(5) | 138.4(4) | | Cl(2)-Bi(1)-S(1) | | 172.0 (1) |
| N(2)-Bi(1)-N(5) | 138.6(3) | | Cl(1)-Bi(1)-S(3) | | 82.9(1) |
| S(1)-Bi(1)-N(6) | 91.2(2) | | Cl(2)-Bi(1)-S(3) | | 92.0(1) |
| S(2)-Bi(1)-N(6) | 69.8(3) | | S(1)-Bi(1)-S(3) | | 96.1(1) |
| N(1)-Bi(1)-N(6) | 130.6(3) | | Cl(1)-Bi(1)-N(5) | | 154.5(2) |
| N(2)-Bi(1)-N(6) | 139.2(4) | | Cl(2)-Bi(1)-N(5) | | 91.1(2) |
| N(5)-Bi(1)-N(6) | 62.6(3) | | S(1)-Bi(1)-N(5) | | 91.1(2) |
| N(1)-Bi(1)-O(13) | 70.6(3) | | S(3)-Bi(1)-N(5) | | 72.7(2) |
| N(5)-Bi(1)-O(13) | 79.2(3) | | | | |
| Cl(1)-Bi(1)-Cl(2) | | 167.6 (1) | | | |
| Cl(1)-Bi(1)-N(1) | | 84.2(2) | | | |
| Cl(2)-Bi(1)-N(1) | | 84.9(2) | | | |
| Cl(1)-Bi(1)-N(2) | | 87.6(2) | | | |
| Cl(2)-Bi(1)-N(2) | | 82.6(2) | | | |
| Cl(1)-Bi(1)-S(1) | | 96.9 (1) | | | |
| Cl(2)-Bi(1)-S(1) | | 87.9 (1) | | | |
| N(1)-Bi(1)-S(1) | | 141.7(2) | | | |

**Table 4.** Selected Bond Angles (deg)

| atom | x | y | z | $U_{equiv.}$ |
|---|---|---|---|---|
| Bi(1) | 2154(1) | 1311(1) | 3030(1) | 44(1) |
| S(1) | 3170(3) | 322(1) | 3060(3) | 57(2) |
| S(2) | 3301(3) | 1862(2) | 2047(3) | 63(2) |
| N(1) | 2802(10) | 2233(5) | 4539(8) | 60(6) |
| N(2) | 4296(8) | 1368(5) | 4567(7) | 48(5) |
| N(3) | 5125(9) | 992(4) | 4534(8) | 49(5) |
| N(4) | 5472(11) | 184(5) | 3747(9) | 58(6) |
| N(5) | 401(8) | 521(4) | 2110(9) | 45(5) |
| N(6) | 884(9) | 1274(4) | 759(7) | 46(5) |
| N(7) | 1094(9) | 1658(4) | 57(8) | 45(5) |
| N(8) | 2348(10) | 2321(5) | -93(9) | 59(6) |
| C(1) | 2050(13) | 2650(6) | 4516(11) | 64(8) |
| C(2) | 2417(15) | 3082(6) | 5366(12) | 72(9) |
| C(3) | 3567(14) | 3069(6) | 6277(11) | 63(8) |
| C(4) | 4351(13) | 2652(6) | 6311(11) | 58(7) |
| C(5) | 3969(12) | 2232(5) | 5422(10) | 48(6) |
| C(6) | 4768(12) | 1766(6) | 5391(10) | 55(7) |
| C(7) | 6087(10) | 1754(5) | 6305(9) | 70(7) |
| C(8) | 4611(12) | 520(6) | 3819(10) | 52(7) |
| C(9) | 6763(12) | 361(6) | 4261(13) | 79(9) |
| C(10) | 7000(18) | 743(8) | 3471(20) | 158(20) |
| C(11) | 6424(28) | 610(11) | 2140(23) | 179(26) |
| C(12) | 5844(37) | 167(16) | 1756(26) | 380(47) |
| C(13) | 5038(20) | -219(8) | 1807(16) | 142(16) |
| C(14) | 5202(14) | -332(6) | 3039(13) | 85(9) |
| C(21) | 142(11) | 130(5) | 2690(10) | 47(6) |
| C(22) | -756(12) | -253(6) | 2241(11) | 61(8) |
| C(23) | -1513(13) | -255(6) | 1059(13) | 69(9) |
| C(24) | -1334(12) | 160(5) | 378(11) | 57(7) |
| C(25) | -383(11) | 540(5) | 906(11) | 44(6) |
| C(26) | -125(10) | 975(5) | 233(10) | 44(6) |
| C(27) | -1040(11) | 1086(5) | -1076(9) | 67(7) |
| C(28) | 2123(11) | 1937(5) | 571(9) | 41(6) |
| C(29) | 1461(13) | 2383(6) | -1381(11) | 81(9) |
| C(30) | 1655(18) | 1939(7) | -2107(13) | 119(12) |
| C(31) | 2867(19) | 1801(12) | -1869(18) | 199(18) |
| C(32) | 3858(19) | 1885(11) | -1094(18) | 215(18) |
| C(33) | 4376(13) | 2201(7) | 26(14) | 96(10) |
| C(34) | 3526(13) | 2602(5) | 234(11) | 70(8) |
| N(11) | 929(11) | 1200(5) | 4795(10) | 63(7) |
| O(11) | 328(8) | 1526(4) | 3938(7) | 77(5) |
| O(12) | 530(12) | 1073(6) | 5443(10) | 140(9) |
| O(13) | 1957(9) | 1002(4) | 4974(7) | 76(5) |

**Table 5.** Atomic Positional Parameters (x $10^4$) and Equivalent Isotropic Displacement Parameters (x $10^3$) for **5**

| atom | x | y | z | $U_{equiv.}$ |
|---|---|---|---|---|
| Bi(1) | 656(1) | 4196(1) | 1821(1) | 46(1) |
| S(1) | 2424(3) | 383(3) | 879(3) | 68(2) |
| S(2) | -651(3) | 6554(3) | 4784(2) | 59(2) |
| S(3) | -569(3) | 2364(3) | 2211(3) | 62(2) |
| S(4) | 2765(3) | 5076(3) | 3610(2) | 63(2) |
| N(1) | -488(10) | 145(8) | 1853(7) | 63(5) |
| C(1) | -18(12) | -837(9) | 1584(10) | 69(7) |
| C(2) | 1171(13) | -832(12) | 2495(10) | 83(8) |
| C(3) | 934(21) | -1062(22) | 3362(15) | 174(19) |
| C(4) | 176(23) | -639(19) | 3741(15) | 150(19) |
| C(5) | -1039(19) | -566(25) | 3174(15) | 231(24) |
| C(6) | -1477(13) | -198(11) | 2236(12) | 87(9) |
| N(2) | 3097(8) | 6361(8) | 5434(7) | 57(5) |
| C(7) | 2789(11) | 7082(10) | 6136(9) | 65(7) |
| C(8) | 3025(13) | 8356(11) | 6103(11) | 80(8) |
| C(9) | 4291(22) | 9019(16) | 6108(21) | 197(24) |
| C(10) | 4998(18) | 8601(19) | 5708(20) | 170(19) |
| C(11) | 5389(13) | 7552(15) | 5713(13) | 106(10) |
| C(12) | 4531(11) | 6485(11) | 5848(10) | 73(7) |
| C(13) | 3581(15) | 109(13) | 577(12) | 95(10) |
| C(14) | 4440(15) | 1136(14) | 581(12) | 103(11) |
| C(15) | 4179(13) | 2201(12) | 820(11) | 81(8) |
| C(16) | 3072(10) | 1918(10) | 1003(8) | 56(6) |
| C(17) | -1944(11) | 6882(11) | 4867(10) | 67(7) |
| C(18) | -3016(13) | 6508(12) | 3900(11) | 81(9) |
| C(19) | -2806(11) | 5942(11) | 3092(10) | 68(7) |
| C(20) | -1560(10) | 5895(9) | 3442(8) | 50(5) |
| N(3) | 1003(8) | 1475(7) | 1553(6) | 49(4) |
| N(4) | 1546(8) | 2623(7) | 1528(6) | 45(4) |
| N(5) | 1014(8) | 5873(7) | 4113(7) | 49(4) |
| N(6) | 103(8) | 5293(7) | 3065(7) | 47(4) |
| C(21) | -1070(10) | 5328(9) | 2778(8) | 48(5) |
| C(22) | -2058(10) | 4722(11) | 1633(9) | 72(7) |
| C(23) | 2502(10) | 2802(9) | 1261(8) | 51(5) |
| C(24) | 3123(10) | 4078(9) | 1242(9) | 65(6) |
| C(25) | 58(10) | 1282(9) | 1836(8) | 48(5) |
| C(26) | 2179(10) | 5810(9) | 4402(8) | 47(5) |
| N(11) | 1084(11) | 6595(8) | 828(8) | 63(6) |
| O(11) | 1931(9) | 6511(8) | 1631(7) | 91(5) |
| O(12) | 1476(9) | 7131(8) | 292(7) | 93(6) |
| O(13) | -118(9) | 6144(8) | 571(7) | 86(5) |
| C(27) | 4171(16) | 7152(17) | 889(15) | 124(12) |
| C(28) | 4949(16) | 7687(18) | 1862(17) | 147(14) |

**Table 6.** Atomic Positional Parameters (x $10^4$) and Equivalent Isotropic Displacement Parameters (x $10^3$) for **9**.

| atom | x | y | z | $U_{equiv.}$ |
|---|---|---|---|---|
| Bi(1) | 1361(1) | 1020(1) | 4849(1) | 31(1) |
| Cl(1) | 3009(3) | 2129(3) | 5923(2) | 64(1) |
| Cl(2) | -127(3) | 29(2) | 3372(2) | 43(1) |
| C(1) | 2009(11) | 2581(7) | 3225(6) | 33(3) |
| C(2) | 3365(11) | 2010(7) | 3227(6) | 33(3) |
| N(1) | 3402(9) | 1184(7) | 3727(5) | 38(3) |
| C(3) | 4578(12) | 618(9) | 3747(7) | 52(4) |
| C(4) | 5813(12) | 851(10) | 3249(8) | 60(5) |
| C(5) | 5822(13) | 1680(10) | 2747(7) | 60(5) |
| C(6) | 4599(12) | 2265(8) | 2725(6) | 43(4) |
| C(7) | 1752(13) | 3401(7) | 2572(6) | 53(4) |
| N(2) | 1011(9) | 2322(6) | 3805(5) | 35(3) |
| N(3) | -257(9) | 2862(6) | 3774(5) | 37(3) |
| C(8) | -1283(11) | 2646(7) | 4378(6) | 31(3) |
| S(1) | -1166(3) | 1753(2) | 5243(2) | 37(1) |
| N(4) | -2547(9) | 3159(6) | 4295(5) | 38(3) |
| C(9) | -2652(12) | 3984(8) | 3653(7) | 48(4) |
| C(10) | -1992(14) | 4893(9) | 4089(9) | 65(5) |
| C(11) | -2293(20) | 5100(11) | 5086(9) | 94(7) |
| C(12) | -2596(30) | 4521(14) | 5739(11) | 241(18) |
| C(13) | -3426(14) | 3668(8) | 5870(7) | 55(5) |
| C(14) | -3728(11) | 3070(8) | 4960(6) | 43(4) |

**Table 7.** Atomic Positional Parameters (x $10^4$) and Equivalent Isotropic Displacement Parameters (x $10^3$) for **15**.

| atom | x | y | z | $U_{equiv.}$ |
|---|---|---|---|---|
| Bi(1) | 1963(1) | 501(1) | 3961(1) | 48(1) |
| Cl(1) | 2149(3) | 839(3) | 5773(2) | 71(1) |
| Cl(2) | 2071(3) | 2549(3) | 3298(3) | 85(2) |
| S(1) | 1426(2) | -1687(2) | 4832(2) | 64(1) |
| C(1) | 2671(9) | -3064(8) | 4133(7) | 53(5) |
| N(1) | 3888(7) | -3458(7) | 4104(6) | 55(4) |
| N(2) | 4051(8) | -2704(7) | 4718(6) | 56(4) |
| C(2) | 5162(9) | -2934(9) | 4797(8) | 56(5) |
| C(3) | 5184(10) | -2016(9) | 5500(8) | 72(6) |
| C(4) | 6339(10) | -3997(9) | 4239(8) | 61(5) |
| C(5) | 7550(11) | -4254(11) | 4309(10) | 77(6) |
| C(6) | 8478(12) | -5356(13) | 3682(11) | 102(8) |
| C(7) | 8026(13) | -5906(12) | 3151(11) | 96(8) |
| S(2) | 6448(3) | -5120(3) | 3397(3) | 77(2) |
| N(3) | 2426(8) | -3841(7) | 3618(6) | 59(4) |
| C(8) | 1118(11) | -3623(11) | 3631(9) | 80(7) |
| C(9) | 1138(18) | -3608(21) | 2604(15) | 225(19) |
| C(10) | 2163(25) | -3806(18) | 1580(15) | 170(20) |
| C(11) | 3506(22) | -4309(22) | 1272(16) | 263(21) |
| C(12) | 4260(14) | -4756(13) | 1857(11) | 105(8) |
| C(13) | 3496(11) | -4930(9) | 2968(9) | 71(6) |
| S(3) | 4460(2) | -445(2) | 3221(2) | 58(1) |

| | | | | |
|---|---|---|---|---|
| C(14) | 4925(10) | -989(11) | 1879(9) | 82(6) |
| N(4) | 4149(8) | -962(9) | 1412(7) | 78(5) |
| N(5) | 2806(8) | -447(7) | 2055(6) | 58(4) |
| C(15) | 2028(9) | -424(9) | 1604(7) | 55(5) |
| C(16) | 565(10) | 186(12) | 2254(8) | 85(6) |
| C(17) | 2435(11) | -959(9) | 513(8) | 65(5) |
| C(18) | 1597(13) | -1084(11) | 83(9) | 85(7) |
| C(19) | 2235(18) | -1595(14) | -985(13) | 122(11) |
| C(20) | 3547(20) | -1898(15) | -1353(11) | 146(12) |
| S(4) | 4066(4) | -1520(4) | -451(3) | 102(2) |
| N(6) | 6213(10) | -1488(14) | 1277(9) | 148(8) |
| C(21) | 6763(17) | -1984(17) | 16(14) | 152(7) |
| C(22) | 8158(24) | -2818(22) | -519(20) | 246(13) |
| C(23) | 7970(34) | -3650(31) | 264(27) | 411(26) |
| C(24) | 8832(23) | -3374(20) | 925(18) | 210(10) |
| C(25) | 7833(21) | -2511(17) | 1819(16) | 174(8) |
| C(26) | 7289(16) | -1336(15) | 1613(14) | 132(6) |

**Table 8.** Atomic Positional Parameters (x $10^4$) and Equivalent Isotropic Displacement Parameters (x $10^3$) for **18**.

**Results and Discussion**

Reaction of bismuth(III)chloride ($BiCl_3$) or bismuth(III)nitrate $Bi(NO_3)_3$ 5 $H_2O$ with thiosemicarbazones and dithiocarbazonic acid methylester derivatives forms bismuth(III)complexes with different molecular structures and stoichiometrical content. Therefore the synthesized bismuth compounds are divided into five groups named D - H. It was possible to resolve the molecular structures of four representative complexes (5, 9, 15, 18) by means of crystal structure analysis.

In the bismuth complexes of group D (1-8), two tridentate ligands and one nitrate are coordinated to the bismuth(III) central atom, the latter via one of its oxygen atoms. In a keto enol tautomerism, the thioketo group is converted into a thiolate group while the proton of the H-N(2) group is split off. Owing to the additional C = N double bond, a conjugated double binding system is formed in the ligand, which contributes considerably to the stabilization of the complexes. Scheme 2 displays the thioketo-enol tautomerism and the complexation possibilities of the pyridine ring containing ligands.

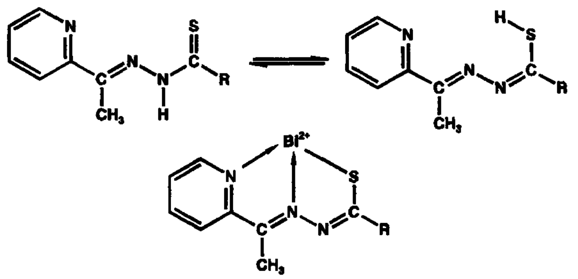

**Scheme 2.** Thioketo-enol tautomerism and complexation possibilities of thiosemicarbazones containing a pyridine heterocycle

The ligands are bound, as tridentate ligands, to the central atom through the nitrogen atom of the pyridine ring, the nitrogen atom of the C = N double bond and the sulfur atom of the thiolate group. The non-bound thiosemicarbazone and dithiocarbazonic acid methylester derivatives can have different stereoisomeric forms (e.g. cis-trans-isomerism with respect to the C = N double bond). Therefore, the $^{1}$H NMR spectra of the free ligands show several signals for the same proton. In the $^{1}$H NMR spectra of the bismuth(III) complexes of type D there is, in each case, exactly one signal for the aromatic and magnetically equivalent protons; i.e., the bound ligand has exactly the stereoisomeric form that has been mentioned above. For the four protons of the pyridine ring H-7 and H-10, two doublets are observed and for H-8 and H-9 one doublet in the range between 7.5 and 9.0 ppm. The pyridine nitrogen binds through the free electron pair. The decreased electron density in the ring system effects a deshielding of the protons and thus a shift to low field by 0.1 - 0.4 ppm. This downfield shift can be observed in the complexes of group D and F.

The structure of compound **5**, a typical representative of the complex group D, can be described as follows. Two deprotonated molecules of **1 g** are configurated to the bismuth(III) cation as tridentate ligands. Except for the hexamethyleneimine ring, the ligand lies on the same plane. The bismuth atom is seven coordinated, as shown in Figure 1. The best description appears to be that of a distorted trigonal dodecahedron with one position occupied by the lone pair of the bismuth atom. Due to this, the Bi(1)-S(2) bond distance near to this electron pair is somewhat longer than the Bi(1)-S(1) bond distance. All of the calculated Bi-N distances differ from each other, the distances between the central bismuth atom and the N-atom of the pyridine rings (N(1), N(5)) are longer than the distances between Bi(1) and the C = N bounded N(2) and N(6). The bonding distance between Bi(1) and O(13) (273.1 pm) is distinctly smaller than the sum of their van der Waals radii (360 pm) and should be treated as polar interactions.[25-27] In the FD mass spectrum, a peak was found at m/z = 759, which corresponds to the fragment $[(Lig)_2Bi^+]$, i.e. the nitrate ion was split off. The molar conductivity $\Lambda_M$ of **5** measured in DMF is 62.3 [$Scm^2mol^{-1}$]. All bismuth complexes of group D show molar conductivities in the range between 62-75 [$Scm^2mol^{-1}$] and therefore behave as 1:1 electrolytes.[28]

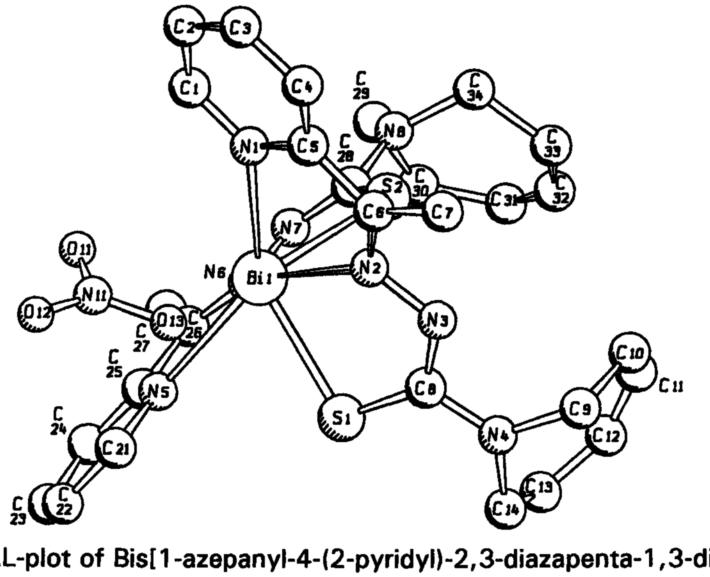

**Figure 1.** SCHAKAL-plot of Bis[1-azepanyl-4-(2-pyridyl)-2,3-diazapenta-1,3-diene-1-thiolato-N', $N^3$,S]bismuth(III) nitrate (**5**)

In the bismuth complexes of group E each time two thiosemicarbazone or dithiocarbazonic acid methylester derivatives are coordinated to the bismuth(III) central atom as bidentate ligands via the N(3) atom and the S atom of the thiolate group. There is no coordinative bond between the sulfur atom in the thiophene ring or the oxygen atom in the furan ring and the bismuth central atom. The $^1H$ NMR spectra of the bismuth complexes from group E show one doublet for the ring protons H-7 and H-9 and a doublet of doublets for the proton H-8 in the range between 6.5-8.1 ppm. In contrast to the free ligands, the shift of the signals is negligible. Molar conductivities $\Lambda_M$, measured in DMF, lie in the range of 47.6-60.6 [$Scm^2mol^{-1}$] and prove that this complex behaves as a 1:1 electrolyte.[28]

Compound 9 is assigned to complex type E, its structure is given in Figure 2. Here, two molecules of 1 k, deprotonated in positions N(4) and N(6), are coordinated to the bismuth(III) central atom as bidentate ligands. The complex bonds are formed by the sulfur atoms of the thiolate groups S(3) and S(4) and by the nitrogen atoms N(4) and N(6). With the exception of the hexamethyleneimino ring, the whole ligand lies within a single plane. The structure of the complex can best be described as a distorted trigonal antiprism, while the central atom is five-coordinated. The two triangular faces are formed by the atoms S(4), N(6), O(11) and S(3), N(4) and the lone pair of the central atom. The two chelate rings are almost perpendicular to each other. The Bi(1)-O(11) distance of 289 pm is considered to be an intermolecular interaction, as mentioned before in compound 5. The methanol molecules required for crystal formation can be found in the gaps of the crystal lattice. In the FD mass spectrum, a peak is obtained at m/z = 769 for the fragment [$(Lig)_2Bi^+$].[29] This is determined by deducting the mass for the methanol molecule and a nitrate ion from the molar peak.

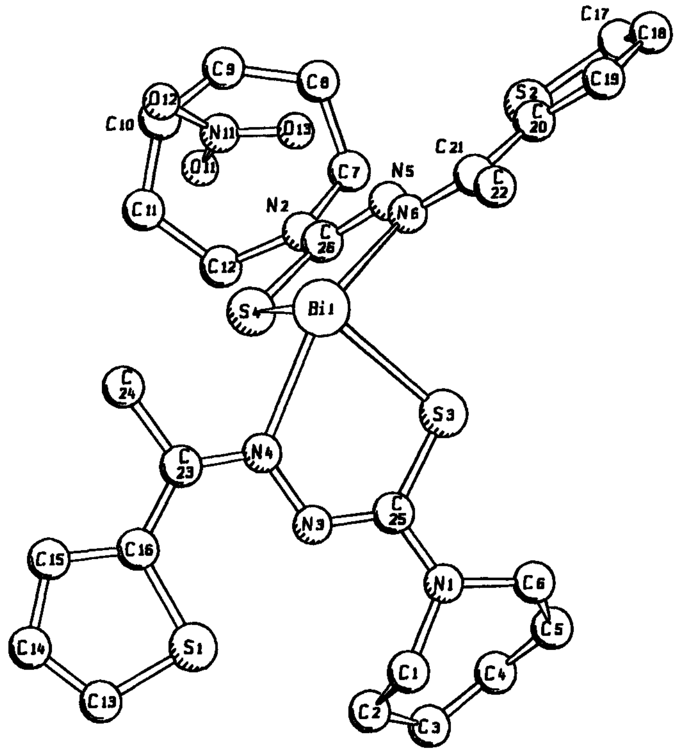

Figure 2. SCHAKAL plot of Bis[1-azepanyl-4-(2-thienyl)-2,3-diazapenta-1,3-diene-1-thiolato-$N^3$,S]bismuth(III) nitrate (9)

The complexes of group F are dimeric bismuth(III) complexes. The two bismuth central atoms are bridged via two chlorine atoms. The tridentate chelate ligands are perpendicular to the $Bi_2Cl_2$-plane and can have a parallel or antiparallel arrangement. The proton of the N(2)-H group shows a singlet $^1H$ NMR signal, which can be observed in the range between 9.5 and 11.5 ppm in the case of the free ligands, it disappears completely in the case of the bismuth complexes of groups D and F. In the $^1H$ NMR spectra, most of the bismuth(III) complexes of group F show a second, weak signal for the protons H-10 and H-13 and in some cases also for the protons H-9 and H-14. This additional signal is caused by the different possibilities in which the tridentate ligand can bind to the bismuth atom, as shown in Scheme 2.

Complex **15** is an example of a dimeric bismuth(III) complex of group F. Its structure, as shown in Figure 3, can be described as two six-coordinated bismuth atoms, which are bound together via two bridging chlorine atoms.[30]

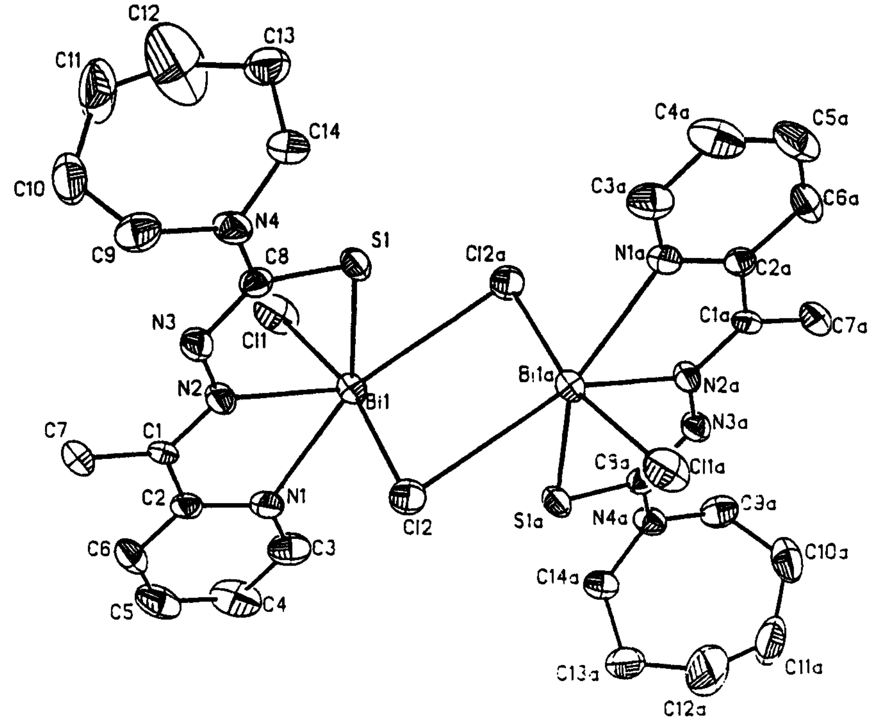

**Figure 3.** ORTEP plot of the dimeric unit of Di-$\mu$-chlorobis[1-azepanyl-4-(2-pyridyl)-2,3-diazapenta-1,3-diene-1-thiolato-N',$N^3$,S-chloro]dibismuth(III) (**15**)

The two bismuth atoms Bi(1) and Bi(1a) and the two bridging chlorine atoms Cl(2) and Cl(2a) form the $Bi_2Cl_2$ plane. The non-bridging chlorine atoms Cl(1), Cl(1a) almost lie in the $Bi_2Cl_2$ plane. The greater bond length Bi(1)-Cl(2) of 279.1 pm in comparison to 258.5 pm in the case of Bi(1)-Cl(1) is an indication of the bridging function of the Cl(2) atom. The Bi(1)-Cl(2a) distance was determined as 316.2 pm. This is a relatively great bond length for a Bi-Cl bond but can be considered as a covalent bond.[31] The two tridentate ligand molecules coordinate via the same atoms as shown in complex **5**. In addition, they form two parallel planes, which are perpendicular to the $Bi_2Cl_2$ plane. With regard to the center of the Bi(1)-Bi(2) axis they are central point symmetrical, i.e. one pyridine ring lies above and the other beneath the $Bi_2Cl_2$ plane.

The FD mass spectrum shows a typical isotope distribution for three chlorine atoms at m/z = 1073, 1075, 1077, 1079, which corresponds to the fragment $[(Lig)_2Bi_2Cl_3^+]$, i.e. a chlorine atom was split off. The molar conductivity $\Lambda_M$ of **15**, measured in DMF, is 8.6 [$Scm^2mol^{-1}$]. All bismuth complexes of group F show molar conductivities in the range between 7.5-9.6 [$Scm^2mol^{-1}$] and are therefore assigned to the neutral complexes.[28]

It was not possible to obtain chloro-bridged dimeric bismuth(III) complexes from the ligands **1 b** and **1 c**, which are not alkylated at the N(12) atom. These two complexes are assigned to group G, where three chlorine atoms and a bidentate ligand are coordinated to the bismuth(III) central atom. The bidentate ligand is bound to the central atom through the N(3) atom and the sulfur atom of the thioketo group. It can be seen from the $^1H$ NMR spectra that the N(2)-H group has the deprotonated form. For the N(2)-H proton, singulets are observed at 11.78 ppm (**19**) and at 11.35 ppm (**20**). If the $^{13}C$ NMR spectrum of **1 b** is compared with the $^{13}C$ NMR spectra of the bismuth complexes **1** and **19**, synthesized with these ligands, it can be demonstrated that the ligand is bound to the bismuth(III) central atom in different ways. While the carbon signals in complex **19** show only a minor shift against the free ligand, they show markedly changed chemical shifts in complex **1**. The complex bond to the N(3) atom, which is formed during the complexation of **1 b** to the bismuth(III) cation, effects a polarisation of the C=N azomethine bond. Due to the reduced electron density, the C(4) signal is shifted by 5 ppm to the lower field. The shift into the high field of the C(1) signal is caused by the keto-enol tautomerism at the C(1) atom. The signals of the ring carbon atoms show a marked tendency towards the lower field. Yet our attempts to resolve the molecular structure of this group of bismuth complexes by crystal structure analyses have been unsuccessful.

On analysis of the reaction product of ligand **1 k** and bismuth(III) chloride, we were surprised to obtain a complex of a fifth group H, the structure of which differs from the other four groups in several ways.

The structure of **18** is completely different from the structures of the bismuth(III) complexes discussed so far. The bismuth central atom is coordinated with two ligands of **1 k**, which are bound in different ways. One of the two ligand molecules is deprotonated. This ligand is bound to the central atom via the sulfur atom S(3) of the thiolate group and the N(5) atom. The thiophene ring is cis-configurated with regard to the N(5)=C(15) bond, with the result that an interaction of S(4) with Bi(1) is not possible. The second molecule of **1 k** is not deprotonated. This ligand is bound to the bismuth(III) cation merely via the sulfur atom S(1) of the thioketo group. The value for the distance between Bi(1) and S(1) of 299.2 pm corresponds to an intermolecular interaction and not to a covalent bond. The best description of the coordination sphere of the bismuth atom is that of a distorted square bipyramidal polyhedron. The square face is formed by the atoms S(3), N(5), Cl(1), the lone pair and the bismuth atom within. The axial positions are occupied by the atoms S(1) and Cl(2). The bond angle between S(1), Bi(1) and Cl(2) differs by about eight degrees from the value determined for a regular square bipyramidal polyhedron of 180 degrees. In the $^1H$ NMR spectrum, a singulet can be observed at 9.42 ppm, which is caused by the proton of the N(2)-H group of the protonated ligand. For the protons of the pyridine rings and the H-5 protons, three different signals are mostly found. The different signals can be produced by the possible Z,E isomerism within the protonated ligand on the one hand and by the difference in the binding of the ligands to the bismuth central atom on the other. The FD mass spectrum shows two signals at m/z = 804, 806 with the typical isotope distribution for one chlorine atom. This fragment, $[(Lig)_2BiCl^+]$, is obtained when deducting the mass of one HCl (X = 36) from the molar mass. A further signal at m/z = 769 is assigned to the fragment $[(HMTH)_2Bi^+]$. The molar conductivity of 3.0 $[Scm^2mol^{-1}]$ in DMF shows that complex 18 is a neutral complex.[28]

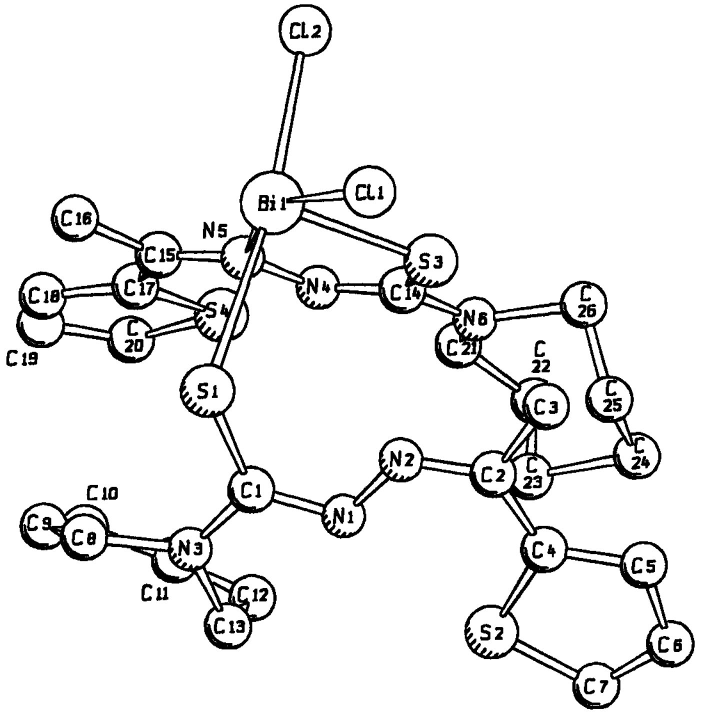

Figure 4. SCHAKAL plot of [1-azepanyl-4-(2-thienyl)-2,3-diazapenta-1,3-diene-1-thiolato-$N^3$,S]-[1-azepanyl-4-(2-thienyl)-2,3-diazapent-3-ene-1-thiolketo-S]dichlorobismuth(III) (18)

There seems to be a relationship between the molecular structure of bismuth(III) compounds, their solubility and their biological activity against *H. pylori* bacteria. In our in vitro experiments, the dimeric bichlorobridged complexes are less soluble and less active than the monomeric species, obtained by reaction of bismuth(III) nitrate and the same thiosemicarbazone that was used to synthesize the dimeric compound. It would be interesting if these tendencies could be observed in studies at gastrointestinal pH values. Therefore further experiments concerning the stability of the new compounds at 37 °C and pH 1 (stomach, mucosa) or pH 10 (intestine) must be carried out.

### ACKNOWLEDGEMENTS

This work has been supported by the Deutsche Forschungsgemeinschaft, DFG, Bonn (FRG).

### REFERENCES

(1) Warren, J. R.; Marshall, B. J. *Lancet* 1983, I, 1273; ibid. 1984, 1311.

(2) Marshall, B. J.; Amstrong, J.; McGeChie, D.; Glancy, R. *Med. J. Aust.* 1985, **152**, 436.

(3) Morris, A.; Nickolsen, G. *Am. J. Gastroenterol.* 1987, **82**, 192.

(4) Malfertheiner, P. *Campylobacter pylori. Neue Aspekte bei chronischer Gastritis und peptischem Ulcus*, Unas Verlag Aachen, Germany, 1988.

(5) Register collection *Scand. J. Gastroenterol. Suppl.* 1991, **187**, 1-107.

(6) Reedijk, J.; Asato, E.; Driessen, W. L.; de Graaff, R. A. G.; Hulsbergen, B. F. *Inorg. Chem.* 1991, **30**, 4210.

(7) Herrmann, W. A.; Herdtweck, E.; Pajdla, L. *Inorg. Chem.* 1991, **30**, 2579.

(8) Domagk, G.; Behnisch, R.; Mietzsch, F.; Schmidt, H. *Naturwissenschaften* 1946, **33**, 315.

(9) Easmon, J.; Heinisch, G.; Holzer, W.; Rosenwirth, B. *J. Med. Chem.* 1992, **35**, 3288-3296.

(10) Klayman, D. L.; Bartosevich, J. F.; Scovill, J. P.; Mason, C. J. *J. Med. Chem.* 1979, **22**, 855, 1367; ibid. 1982, **25**, 1261; ibid. 1983, **26**, 35.

(11) Klayman, D. L.; Bartosevich, J. F.; Scovill, J. P.; Mason, C. J. *Eur. J. Med. Chem.* 1981, **16**, 317.

(12) For a review article see Douglas X. West et al. *Coord. Chem. Rev.* 1993, **123**, 49-71.

(13) Reddy, P. S. N.; Agarwala, B. V. *Synth. React. Inorg. Met.-Org. Chem.* 1987, **17**, 585.

(14) Singh, D.; Singh, R. V. *Phosphorus, Sulfur, and Silicon* 1991, **61**, 57-64.

(15) Singh, K.; Singh, R.V., Tandon, J. P. *J. Prakt. Chem.* 1988, **330**, 621-625.

(16) Singh, K.; Tandon, J. P. *Monatsh. Chem.* 1992, **123**, 315-319.

(17) Riddick, J. A.; Bunger, W. B. *Organic Solvents: Physical Properties and Methods of Purification*, 3rd ed.; Wiley, New York, 1970.

(18) Anthoni, U.; Larsen, C.; Nielsen, P. H. *Acta. Chem Scand.* 1968, **22**, 1898.

(19) Anderson, F. E.; Duca, C. J.; Scudi, J. V. *J. Am. Chem. Soc.* 1951, **73**, 4967.

(20) Xia, H.; Daw, M. A.; Sant, S.; Beattie, S.; Keane, C. T.; O'Morain, C. *Eur. J .Gastroenterol. Hepatol.* 1993, **5**, 141-144.

(21) Labenz, J.; Gyenes, E.; Rühl, G. H.; Börsch, G. *Am. J. Gastroenterol.* 1993, **88**, 419-495.

(22) DIF4, Version 6.1, program for Siemens-Stoe AEDII Vierkreis diffractometer, Darmstadt, Germany, 1984.

(23) Sheldrick, G. M. *SHELXTL-PLUS-Programm*, Universität Göttingen, Germany, 1988.

(24) *International Tables For X-Ray Crystallography*, The Kynoch Press, Birmingham, England, 1974; Vol. IV.

(25) Herrmann, W. A.; Herdtweck, E.; Scherer, W.; Kiprof, P.; Pajdla, L. *Chem. Ber.* 1993, **126**, 51-56.

(26) Herrmann, W. A.; Herdtweck, E.; Scherer, W.; Kiprof, P.; Pajdla, L. *Chem. Ber.* 1993, **126**, 895-898.

(27) Smith, G.; Reddy, A. N.; Byriel, K.A; Kennard, C. H. L. *Austr. J. Chem.* 1994, **47**, 1413-1418.

(28) Geary, W. J.; *Coord. Chem. Rev.* 1971, 7, 81.
(29) Lig is the abbreviation for ligand molecules.
(30) Due to the central point symmetry, bond lengths and bond angles are only given for one molecule of the dimeric unit.
(31) Müller-Becker, S.; Schneider, J. *Z. Anorg. Allg. Chem.* 1993, 619, 1073.

**Received: July 14, 1995 - Accepted: August 22, 1997 - Accepted in revised camera-ready format: October 3, 1995**

